# Micro-/Nanofibrillated Cellulose-Based Coating Formulations: A Solution for Improving Paper Printing Quality

**DOI:** 10.3390/nano12162853

**Published:** 2022-08-18

**Authors:** Mohit Sharma, Roberto Aguado, Dina Murtinho, Artur J. M. Valente, Paulo J. T. Ferreira

**Affiliations:** 1 University of Coimbra, CIEPQPF, Department of Chemical Engineering, Rua Sílvio Lima, Pólo II–Pinhal de Marrocos, 3030-790 Coimbra, Portugal; 2 LEPAMAP-PRODIS Research Group, University of Girona, M Aurèlia Capmany 61, 17003 Girona, Spain; 3 University of Coimbra, CQC, Department of Chemistry, Rua Larga, 3004-535 Coimbra, Portugal

**Keywords:** betaine hydrochloride, micro-/nanofibrillated cellulose, starch betainate, Pluronics, printing quality

## Abstract

The use of micro-/nanofibrillated celluloses (M/NFCs) is often considered for the enhancement of paper properties, while it is still challenging to use them in lower weight gain coatings. This work explores how they might be used on the paper surface to improve the printing quality. In this regard, M/NFCs were produced using different pre-treatment methods, including mechanical (m-MFC), enzymatic (e-MFC), TEMPO-mediated oxidation (t-NFC) and cationization (c-NFC), and uniform coating formulations were developed through the cooking of starch and M/NFCs simultaneously. The formulations, at 6–8% of total solid concentration, were applied to the paper surface by roll coating, resulting in a dry coating weight of 1.5 to 3 g/m${}^{2}$. Besides M/NFCs, other components such as starch betainate (a cationic starch ester; SB), Pluronics^®^ (a triblock co-polymer), precipitated calcium carbonate (PCC) and betaine hydrochloride (BetHCl) were also used in the M/NFC-based coating formulations to observe their combined influence on the printing quality. The presence of M/NFCs improved the paper printing quality, which was further enhanced by the increase in cationic charge density due to the presence of BetHCl/SB, and also by Pluronics^®^. The cationic charge of c-NFC was also found to be effective for improving the gamut area and optical density of coated papers, whereas whiteness was often reduced due to the quenching of the brightening agent. BetHCl, on the other hand, improved the printing quality of the coated papers, even though it was more effective when combined with M/NFCs, PCC and Pluronics^®^, and also helped to retain paper whiteness.

## 1. Introduction

Paper industries are constantly seeking competitive advantages and, at the same time, exploring sustainable approaches by introducing renewable and biodegradable alternatives for synthetic chemicals in the wet end of the paper machine, in the size press or at any other papermaking stages. In this context, micro-/nanofibrillated celluloses (M/NFCs) constitute a promising alternative that has the potential to be used in paper applications such as filler flocculation and paper reinforcement [1,2]. However, because of their poor compatibility with the wet-end additives and delayed dewatering, their acceptability in these industries is still challenging. As an alternative, M/NFCs coatings have recently emerged to avoid such disruption [3,4], while also improving barrier [5,6], strength [7], and printing quality properties [8,9,10].

Highly concentrated and highly viscous M/NFCs suspensions, by themselves, are unable to produce homogeneous coating layers [11,12], making them ineffective for improving paper surface properties, including printing quality [10]. Depending on the application, previous chemical modifications of cellulose [13] or its combination with other polymers [14] may be helpful. For the preparation of uniform coating formulations, a strategy reported in the literature is based on combining M/NFCs with sizing agents and binders such as starch, poly-vinyl alcohol (PVA), alkyl ketene dimer (AKD), clay, and modified calcium carbonate. M/NFCs promote the retention of ink pigments on the paper surface, while sizing agents such as starch, PVA and AKD (which decrease the wettability of the surface) limit the penetration of the ink carrier solvent, resulting in improved paper printing quality. Moreover, the addition of mineral pigments enhances the smoothness of coated papers’ surface, resulting in improved print density for off-set and inkjet printing [8,10,15,16,17].

Besides the works cited above, little research has focused on using M/NFCs to improve the inkjet printing quality of office papers, which requires a low weight gain coating and an excellent uniformity of coating formulations. For this purpose, the present study aims at using M/NFCs in combination with starch betainate (SB), obtained by a previously published novel approach [18] and Pluronic^®^ block copolymer (P123). To the best of our knowledge; this is the first article reporting the combination of M/NFCs with these two components. Recent research indicates that the use of cationic starch ether/ester in combination with Pluronics improves the inkjet printing quality of office paper, since a positive surface charge exhibits enhanced ink hold properties and establishes synergetic interactions with non-ionic tri-block surfactant polymers. Moreover, the cationic starch ester, SB, has certain advantages over the conventional cationic starch ether, at least in terms of maintaining the whiteness of coated paper in presence of a sulfonated optical brightening agent (OBA) [19]. A hypothesis that is worth considering is whether the direct use on paper of the zwitterionic amino acid used to synthesize SB, i.e., betaine, which is commercially available as betaine hydrochloride (BetHCl), could also attain positive results, saving the costs of the synthesis process.

This study illustrates the effect of M/NFCs on paper printing quality, where microfibrillated celluloses were produced following mechanical and enzymatic pre-treatments. In parallel, the treatments preceding the generation of nanofibrillated celluloses were TEMPO-mediated oxidation towards carboxyl groups, on one hand, and periodate-mediated oxidation (towards carbonyl groups) followed by cationization (quaternary ammonium groups attached via imine bonds), on the other. Further, these M/NFCs were used in combination with native starch, SB, BetHCl, Pluronics (P123), and precipitated calcium carbonate (PCC), to observe their effect on key parameters of printing quality such as gamut area (GA), optical density (OD), print-through (PT) and inter-color bleed (ITCB). Moreover, this paper reports an unconventional approach for preparing uniform coating formulations using the aforementioned components: native starch and/or starch betainate were cooked along with M/NFCs in an aqueous medium, to promote favorable non-covalent interactions between starches and cellulose.

## 2. Materials and Methods

### 2.1. Materials

Corn starch, bleached eucalyptus kraft pulp (BEKP), bleached pine kraft pulp (BPKP), α-amylase (in standard buffer solution, pH 5.8), PCC, a hexasulfonated stilbene-based optical brightening agent (OBA) and alkyl ketene dimer (AKD) were of industrial origin. BetHCl (99%) was purchased from Alfa Aesar and used as-is for transesterification and as a coating agent. Pluronics^®^ P123 (MW 5750 g mol^−1^, PEO 30%wt.) was purchased from Sigma-Aldrich. All solvents were purified or dried prior to use, following standard procedures. Other commercially available compounds such as thionyl chloride, methanol and sodium hydroxide were used without further purification.

### 2.2. Production of Micro-/Nanofibrillated Celluloses

Two kinds of MFCs, m-MFC and e-MFC, were produced using mechanical and enzymatic pretreatment, respectively. For the production of e-MFC, BEKP (17 ºSR) was slushed (4.5% consistency) and refined to 25 ºSR. After that, an endoglucanase enzyme pre-treatment, consisting of 0.17 mL of enzyme cocktail per kg of fiber (on a dry weight basis), for 2 h at 50 °C and pH 6.5, was applied. Then, a second refining stage (80 ºSR) was performed before homogenizing at 750 bar using a high-pressure homogenizer (HPH, GEA Niro Soavi, model Panther NS3006L). In the case of m-MFC, BPKP was intensively refined in a disk refiner.

Likewise, two kinds of NFCs, t-NFC and c-NFC, were also produced by two different pretreatment methodologies: TEMPO-mediated oxidation and cationization of cellulose, respectively. The t-NFC was prepared as described elsewhere (Lourenço et al., 2017 [20]). Briefly, BEKP was refined to 4000 rev. in a PFI beater, and NaBr (0.016 g/g of fibers) and tetramethylpiperidine N-oxyl radical (TEMPO) (0.016 g/g of fibers) were added to the pulp mixture, followed by the addition of NaClO solution (11 mmol NaClO/g of fibers) while maintaining the pH at 10 for two hours. Then, oxidized samples were passed twice through the HPH, at 500 bar and 1000 bar, for obtaining t-NFC (COOH content: 1332 µmol/g).

In parallel, the c-NFC was prepared as described elsewhere [21]. Briefly, BEKP (2.5% *w*/*w*) was oxidized using sodium periodate (0.55 mol/mol of AGU) in isopropanol/water (1:9, *v*/*v*) for 4 h at 70 °C. Oxidized BEKP (5%, *w*/*w*), with a degree of substitution (DS) of 0.48, was cationized using Girard’s reagent T (0.7 mol/mol of aldehyde) in aqueous acetic acid (10%, *w*/*w*). The modified pulp was passed twice through the HPH, at 500 bar and at 700 bar, for obtaining c-NFCs with a DS of 0.16. All M/NFC samples were characterized as follows. The nanofibrillation yield is expressed as the weight ratio, on a dry basis, of a diluted sample (0.2%) that did not sediment when centrifuged at 9000 RPM for 30 min [22]. Consequently, those samples that tended to sediment (yield < 20%) were regarded as MFC. In contrast, samples that formed stable dispersions (yield > 90%), in which the particle size was low enough for the Brownian motion to prevail over gravitational effects, were labeled as NFC.

Zeta potential measurements were performed by means of Malvern Panalytical’s Zetasizer Nano ZS90 (Worcestershire, United Kingdom). Intrinsic viscosity values were calculated from the relative viscosity according to the ISO 5351 standard. In turn, the relative viscosity was determined using a size 100 Cannon–Fenske viscometer in a thermostatic bath set at 25 °C. The degree of polymerization was then estimated from the intrinsic viscosity by applying the Mark–Houwink parameters for cellulose [23]. The results are summarized in Table 1.

### 2.3. Production of Starch Betainate

Starch betainate was synthesized using the transesterification of starch with methyl betainate chloride (MeBetCl) in polar aprotic solvents, according to the methodology reported elsewhere [18]. Briefly, 24 g of betaine hydrochloride were first esterified towards MeBetCl, using 11.3 mL of thionyl chloride and 75 mL of methanol, under reflux, for 4 h at 70 °C. MeBetCl was recovered by evaporating methanol, trituration in diethyl ether, and then drying the crude product under high vacuum. Then, 10 g of cooked starch were converted into SB using 20.8 g of MeBetCl in *N*,*N*-dimethylformamide (DMF). Prior to transesterification, cooked starch had been pre-activated in NaOH/ethanol. The reaction was carried out for 24 h at 70 °C. SB was then further precipitated by adding ethanol (alcohol/water > 10, *v*/*v*), vacuum filtered and washed with absolute ethanol, followed by drying at 50 °C. The degree of substitution of this starch derivative, as estimated by ^1^H-NMR spectroscopy, was 0.3.

### 2.4. Preparation of Coating Formulation

**Figure 1:**
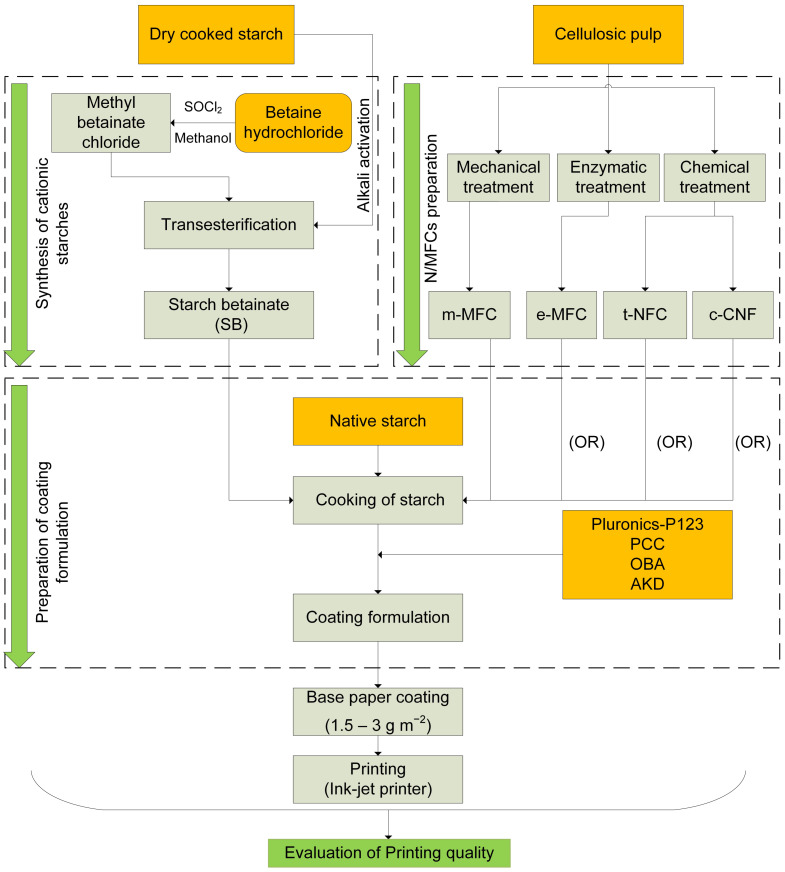
Outline of the experimental procedure.

The aqueous suspensions that were prepared for coating can be divided in three subsections: M/NFC coatings; M/NFC coating with SB and Pluronics; M/NFCs coating with BetHCl and Pluronics. M/NFC was added to the starch solution, cooked together for 5 minutes at 80 °C with α-amylase buffer, and then heated to 90–95 °C, and kept at that temperature for 15 min so that a macroscopically homogeneous dispersion was obtained. Figure 1 depicts a schematic diagram for the preparation of formulations using different coating components.

To study the effect on printing properties, different concentrations of M/NFCs and their combination with starch betainate, Pluronics-P123, PCC, OBA and AKD were chosen and are summarized in Table 2 and Table 3. All percentages express mass of coating component, in each case, per total dry solids weight and all formulations were prepared for achieving 6–8% of total solid concentration. Cooked native starch was always used as a host polymer. The effect of different proportions of M/NFC was studied from 16% to 48%, whereas only the 16% proportion was selected to evaluate the combination of M/NFCs with SB, Pluronics, PCC and other agents (OBA and AKD). The point of addition of each component in the formulations is shown in Figure 1.

Moreover, BetHCl was also added along with the M/NFCs, Pluronics-P123, PCC, OBA and AKD to study their synergetic effect on printing properties. However, a lower concentration of Pluronics (5%), in comparison to the above-mentioned coating formulations of Table 2 and Table 3, was considered, which is still higher than the corresponding critical micelle concentration of Pluronics-P123. A systematic representation of all used concentrations is displayed in Table 4.

### 2.5. Paper Coatings

A Mathis laboratory coater (SVA-IR-B) with an infrared system attached to the applicator bar was used to coat an industrial, calendared and uncoated paper (base paper, BP), produced from BEKP with a basis weight of ≈78 g m^−2^. A 13 mm (diameter) applicator roll was utilized with a velocity of 6 m min^−1^ and intermediate load at both sides to obtain a dry coating weight of 1.5–3.0 g m^−2^ on one side of BP. All coated paper sheets were air-dried at room temperature, while being physically restrained on a metal plate to prevent shrinking. Samples with macroscopically visible defects on their surface were discarded and repeated anew.

The difference between the basis weights (ISO standard 536:1995) of the air-dried coated paper sheet and the respective BP sheet was used to compute the coating weight. All coated papers were kept at controlled conditions of temperature (23 ± 1 °C) and humidity (RH 50 ± 2%) prior to the characterization. Three paper sheets were coated for each formulation and characterized to assess the printing quality.

### 2.6. Evaluation of Printing Qualities

Overall, the printing quality was evaluated as reported elsewhere [19]. Briefly, an inkjet printer (HP Officejet Pro 6230) was used to color-print all coated papers, besides BP as reference. Seven different color spots (black, red, green, blue, cyan, magenta and yellow) were printed, including lines (black lines, yellow lines, black lines on yellow background and yellow lines on black background) and dots (black and magenta). The printed sheets were kept for 4 h under controlled conditions of temperature (23 ± 1 °C) and humidity (RH 50 ± 2%) before any characterization.

An X-Rite Eye One XTreme UV-Cut spectrophotometer was used to quantify the reflectance of six color spots, in this sequence: red, green, blue, cyan, magenta and yellow, after activating the UV light (D50, 2º). The GA is the area of the hexagon framed by these points in the CIELAB two-dimensional color space. The coordinates that locate any point across this space, a* and b*, were obtained from reflectance measurements. Their values on all six points were used to calculate the GA. In addition, CIE L* a* b* coordinates for unprinted white spot and seven color spots (including black color) were used to calculate the gamut volume (GV) of the printed paper sheets.

Other printing properties, such as OD and PT, were evaluated by means of a QEA PIAS-II spectrophotometer with a low-resolution optical module (33 µm/pixel with visual area of 21.3 mm × 16 mm), whose software (PIAS II) is based on the ISO 13660 quality standards. Equation (1) was used to compute the relative OD, which was then corrected by subtracting the OD of a non-printed spot of paper to obtain the absolute OD. The PT of a printed paper required the determination of the CIE L* a* b* coordinates on the opposite side, in contrast with the same coordinates of non-printed area of the same paper sheet. The intensity of the transmitted light from a specific area of each color (black, white, cyan, magenta and yellow) was measured using QEA PIAS-II, and thus PT was determined using Equation (2).

(1) $$OD=lg\frac{Insident\;light}{Transmitted\;light}$$

(2) $$\mathrm{PT}=\sqrt{({\left(L_{p}^{*}-L_{u}^{*}\right)}^{2}+{\left(a_{p}^{*}-a_{u}^{*}\right)}^{2}+{\left(b_{p}^{*}-b_{u}^{*}\right)}^{2}}$$

where L^*^, a^*^ and b^*^ are the CIE chromatic coordinates, and the subscripts *u* and *p* refer to areas of unprinted and back of the printed black spot, respectively.

Furthermore, an additional printing property, inter-color bleed (ITCB), was estimated using a high-resolution module (5 µm/pixel with visual area of 3.2 mm × 2.4 mm) that allowed one to measure the raggedness. Raggedness is directly related to the geometric distortion of the boundary lines, and is given by the standard deviation of the distribution of the distortion of the line adjusted to its ideal limit. The greater the distortion, the worse the ITCB. It was measured for the printed yellow and black lines.

### 2.7. Evaluation of Other Paper Properties

Apparent viscosity at 50 °C of starch-M/NFC coating formulations was measured by means of a Haake rheometer, Model RS1 (Germany), using a plate geometry. A shear stress in a range of 0.5 to 300 Pa was applied. Flow curves were obtained by means of Haake RheoWin 4.20.005 software.

An OCA 20 goniometer (Dataphysics, Germany) was used to assess the static (SWCA) and dynamic contact angle with water (DWCA). The sessile drop method was used to measure the contact angle. A droplet of deionized water (10 µL) was poured over the coated paper surface and after the drop had settled, the resulting angle was measured by fitting the Young–Laplace equation to the drop profile. Bendtsen roughness (ISO 5636-3, 8791-2) and Gurley air permeance (ISO 5636/5) were measured for the coated papers using the respective testers from Frank-PTI. Roughness is quantified by the airflow between a metal head and the paper sample (mL min^−1^), placed on an impermeable surface. In contrast, air permeance refers to the airflow rate through the paper sample (μm Pa^−1^ s^−1^), driven by 1.22 kPa pressure gradient. An Elrepho spectrophotometer (using D65 illumination) was used to measure the whiteness (CIE W D65/10) of the coated papers. For contact angle, Bendtsen roughness, Gurley air permeance and whiteness, the average value and the standard deviation of four independent measures were computed.

## 3. Results and Discussion

### 3.1. Rheology of M/NFCs/Starch-Based Coating Formulations

**Figure 2:**
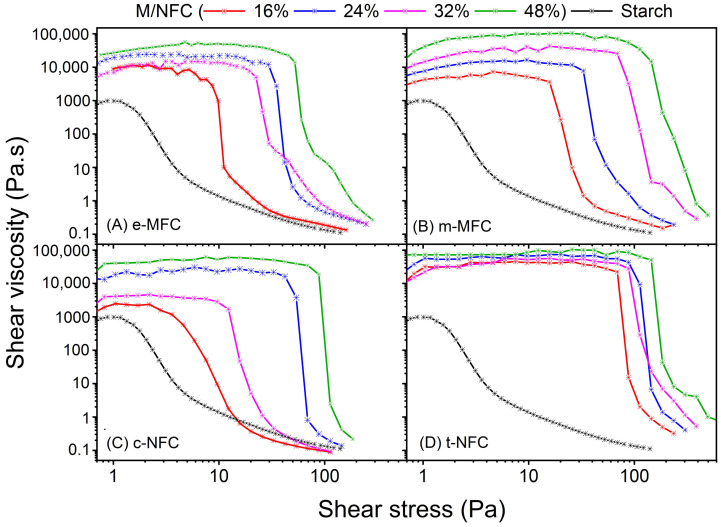
Rheology of micro-/nanofibrillated celluloses-starch based formulations, (**A**) enzymatic produced MFC, (**B**) mechanically produced MFC, (**C**) TEMPO-NFC and (**D**) cationic-NFC for their different concentrations (*w*/*w*.%).

MFCs/starch based formulations showed a shear thinning behavior, in which apparent viscosity decreased as shear rate increased (see Table 5 for apparent viscosity at 10 and 1000 s^−1^ shear rate), which is convenient for paper coating [12]. Figure 2 shows that the shear viscosity increased with the amount of M/NFCs. The formation of a more entangled network of M/NFCs fibrils was enhanced at higher concentration of these fibrils as suggested by the increasing in the viscosity [24]. When compared to formulations with higher concentrations of M/NFCs, the one with 16% M/NFCs showed better conditions for coating because of its lower initial apparent viscosity and yield stress.

Nonetheless, visual inspection demonstrated that MFCs’ concentration up to 32% can be considered for uniform coating. Above that concentration, i.e., 48%, the homogeneity of the formulations was downgraded and a high yield stress is observed. t-NFC-based coating formulations demonstrated the highest shear viscosity and yield stress compared to other M/NFCs at any studied concentration. However, the reduction in apparent viscosity was more abrupt in the case of t-NFC, whose carboxyl content can be related to its rheological behavior [25]. In contrast, c-NFC-based coating formulations substantially reduced the shear viscosity and yield stress at lower concentrations (i.e., 16 and 32%).

### 3.2. Paper Printing Properties

#### 3.2.1. Micro-/Nanofibrillated Celluloses-Starch-Based Coating

**Figure 3:**
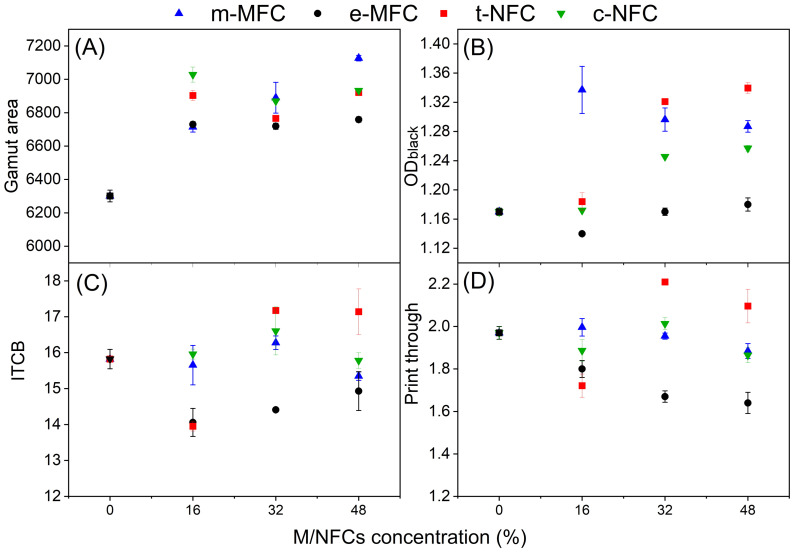
Effect of different concentration of micro-/nanofibrillated celluloses on (**A**) gamut area; (**B**) optical density for black; (**C**) inter color bleed; (**D**) print through.

When comparing the printing quality of mechanically and enzymatically produced MFCs, Figure 3A reveals that GA was increased by roughly 7% from that of starch-coated papers, when the proportion of both MFCs was 16% of the total solids concentration in respective formulations. An additional increase in m-MFC and e-MFC concentrations up to 32% improved the GA by 9.3% and 6.8%, respectively, whilst an increase up to 48% increased the GA by 13.1% and 7.3%, respectively.

Paper coated with m-MFC showed more hydrophilic surfaces than those of e-MFC coatings, as indicated by the static contact angle in Table 6. Therefore, ink holdout properties were particularly enhanced by m-MFC [8]. Although more research is still necessary to fully understand the effects of mechanical stress and endoglucanase-mediated hydrolysis on cellulose, it is generally accepted that the latter targets the amorphous regions [26,27]. Mechanical stress tends to break polymer chains at the middle and to increase their reactivity [28], besides disrupting the structure of the fiber, and thus the higher hydrophilicity that m-MFC produced could be explained by the more severe loss of supramolecular order during the pre-treatment [29].

In the case of NFC-based coatings, Figure 3A reveals that the GA was increased by 9.6 and 11.5% using 16% t-NFC and c-NFC, respectively, also compared to the starch-coated papers. Compared to MFCs, NFCs formulations showed higher GA at lower concentrations. However, there was no substantial improvement when increasing the concentration of these NFCs. Higher concentrations of NFCs, on the other hand, increased PT values, given that the sorption of water-based carrier solvents increases with increasing charge density [30].

Table 6 also shows that the smoothness of M/NFC-coated papers was comparable to that of starch-coated papers. Out of all M/NFCs, paper coated with e-MFC showed the highest smoothness. Likewise, ITCB improved by 11.1, 8.9 and 5.6% when employing 16, 32 and 48% of e-MFC, respectively, whereas other M/NFCs showed no improvement in ITCB (Figure 3C).

Paper sheets coated with M/NFC and starch displayed similar air permeance as those coated with starch alone, hinting that the latter acted as host polymer. The permeance of the uncoated sheets was only slightly greater, 11.3 ± 0.4. Perhaps due to the low coat weight (1.7–2.9 g m^−2^), coatings did not attain high air resistance. However, this was probably convenient for certain printing properties, since the most permeable coating formulation, e-MFC/starch, also improved the PT by 8.6, 15.2, 16.8% using 16, 32, and 48% of e-MFC, respectively, whereas other M/NFCs demonstrated no improvement in PT (Figure 3D).

Figure 3B shows that the highest increase of 14.3% in the optical density of black was obtained with 16% m-MFC-coated papers. An additional increase in m-MFC concentration did not improve the OD (black). At the same time, e-MFC showed no improvement in OD (black) at any used concentration. However, in the case of NFC coatings, 32% t-NFC or c-NFC increased the OD (black) by 12.9 and 6.5%, respectively.

As shown in Table 6, the presence of OBA in formulation with both MFCs and t-NFC coating formulations improved the whiteness of coated papers; however, the presence of c-NFC drastically lowered it. It also decreased as the concentration of c-NFC increased. Similar findings were also reported in our prior investigation with the use of cationic starch ether in the coating formulation, as whiteness was reduced with the addition of said cationic starch [19].

#### 3.2.2. MFCs/Starch, Starch Betainate (SB), Pluronics and PCC-Based Coatings

**Figure 4:**
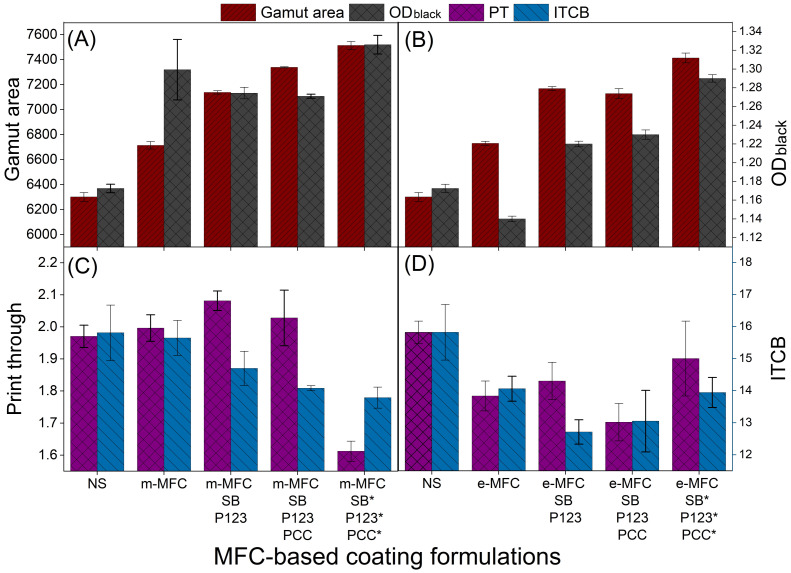
Effect of different concentrations of mechanically produced MFC and enzymatically produced MFC, Starch betainate, P123 and PCC on (**A**,**B**) gamut area and optical density for black; (**C**,**D**) print through and inter color bleed (* 16% component concentration in the formulation).

For the combined coatings, a MFC concentration of 16% was selected because of their suitable rheology for coatings and the desirable paper properties attained, as discussed before. Figure 4 shows the printing properties of papers coated using formulations containing starch, m-MFC, e-MFC, SB, Pluronics and PCC. Two coatings for comparison purposes are also presented: native starch and starch with 16% MFCs as can be observed in Figure 4.

In Figure 4A,B, the GA improved by 6.6% and 6.8% with 16% m-MFC and e-MFC, respectively. Furthermore, SB and Pluronics-P123 (8% of each) increased the GA by 13.3% (m-MFC) and 13.8% (e-MFC) in combination.

GA was improved by 16.5% (with m-MFC) and 13.1% (with e-MFC) with the addition of 8% of PCC. The highest increase in the GA was noticed when 16% of each component was used in the coating formulations: 19.2% for m-MFC and 17.7% for e-MFC. Likewise, Figure 4A,B show that OD (black) was also improved by 11.1, 8.9, 8.6 and 13.3% using m-MFC, m-MFC/SB (8%)/P123 (8%), m-MFC/SB (8%)/P123 (8%)/PCC (8%), m-MFC/SB (16%)/P123 (16%)/PCC (16%), respectively. In case of e-MFC coatings, the OD (black) changed by −2.6, 4.3, 5.1 and 10.3%.

Contrary to the GA and OD, PT demonstrated no improvement upon the addition of SB or P123 in m-MFC-based formulations and as observed from Figure 4C,D. Besides, the addition of PCC lowered the penetration of ink pigments as also detected elsewhere [15] and resulted in lower values of this property. PT improved by 18.2% with 16% of each component, along with m-MFC, whereas ITCB increased by up to 12.8%. This ITCB variation is explained by the fact that cationic sizing reduces the ink feathering due to the electrostatic adsorption of anionic dyes, resulting in quick fixation of ink particles onto the paper surface and preventing their migration [31]. Regarding e-MFC coatings, PT improved with the addition of these e-MFCs; SB, P123 and PCC (8% of each) showed the highest improvement in PT, 12.2%. ITCB followed the same trend as PT, but the highest increase, 19.7%, was achieved using SB and P123 (8% each, in combination with e-MFC).

#### 3.2.3. NFCs/Starch, Starch Betainate (SB), Pluronics and PCC-Based Coatings

**Figure 5:**
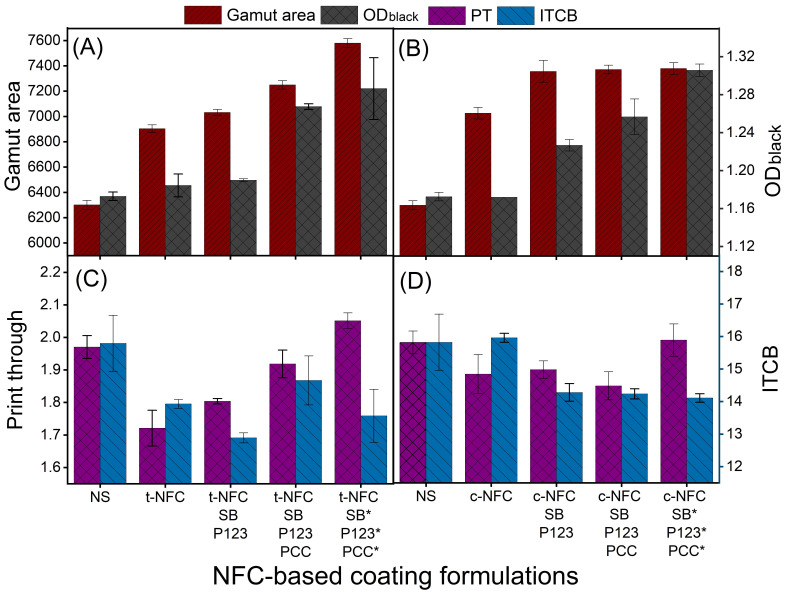
Effect of different concentrations of TEMPO-NFC and cationic-NFC, Starch betainate, P123 and PCC on (**A**,**B**) gamut area and optical density for black; (**C**,**D**) print through and inter color bleed (* 16% component concentration in the formulation).

Similarly to MFCs coatings, a NFC proportion of 16% was chosen; Figure 5 shows the printing properties of paper coated using formulations containing starch, t-NFC, c-NFC, SB, P123 and PCC. Figure 5 additionally presented two coatings for comparison purposes: native starch and 16% NFCs/starch. In Figure 5A,B, GA improved by 9.6% and 11.5% with 16% t-NFC or c-NFC, respectively. Furthermore, SB and P123 (8% of each) increased the GA by 11.6% (t-NFC) and 16.8% (c-NFC) in combination.

GA was improved by 15% (with t-NFC) and 17% (with c-NFC) after an additional 8% of PCC was added. The highest increase in the GA was noticed when 16% of each component was used in the coating formulations, which was 20.3% (t-NFC) and 17.2% (c-NFC). Likewise, Figure 5A,B show that OD (black) was also improved by 1.2, 1.7, 8.3 and 9.9% using t-NFC, t-NFC/SB (8%)/P123 (8%), t-NFC/SB (8%)/P123 (8%)/PCC (8%) and t-NFC/SB (16%)/P123 (16%)/PCC (16%), respectively, and it was 0.2, 4.8, 7.4 and 11.6% (following the same sequence) in the case of c-NFC coatings.

Contrary to the GA and OD, as observed from Figure 5C,D, PT improved by 12.6 and 4.2% using 16% of t-NFC and c-NFC, respectively. However, the further addition of SB, P123 and PCC demonstrated no improvements for both NFC-based formulations. Nonetheless, ITCB improved with the addition of these components and demonstrated a maximum improvement of 18.4% using SB and P123 (8% of each) for t-NFC coatings. Regarding c-NFC coatings, a highest increase of 10.8% was achieved in ITCB using SB, P123 and PCC (16% each, in combination with c-NFC).

Table 6 and Table 7 shows that the whiteness of coated papers was reduced with the use of c-NFCs because of their positively charged, electron-withdrawing quaternary ammonium groups, as they quenched the OBA which used to improve the whiteness of papers. This OBA is a sulfonated derivative of stilbene, and thus the interaction between its negatively-charged functional groups and quaternary ammonium groups may induce aggregation and/or electron transfer-mediated quenching. The whiteness was not affected with the use of other M/NFCs, SB or P123.

#### 3.2.4. Coating Formulations with BetHCl

In order to reduce the cost of coating formulations, instead of cationizing the starch, BetHCl was directly added to the rest of the components of the coating suspension. These experiments also implied a lower concentration of P123 (5%) and higher total solids concentration (10%, *w*/*w*).

**Figure 6:**
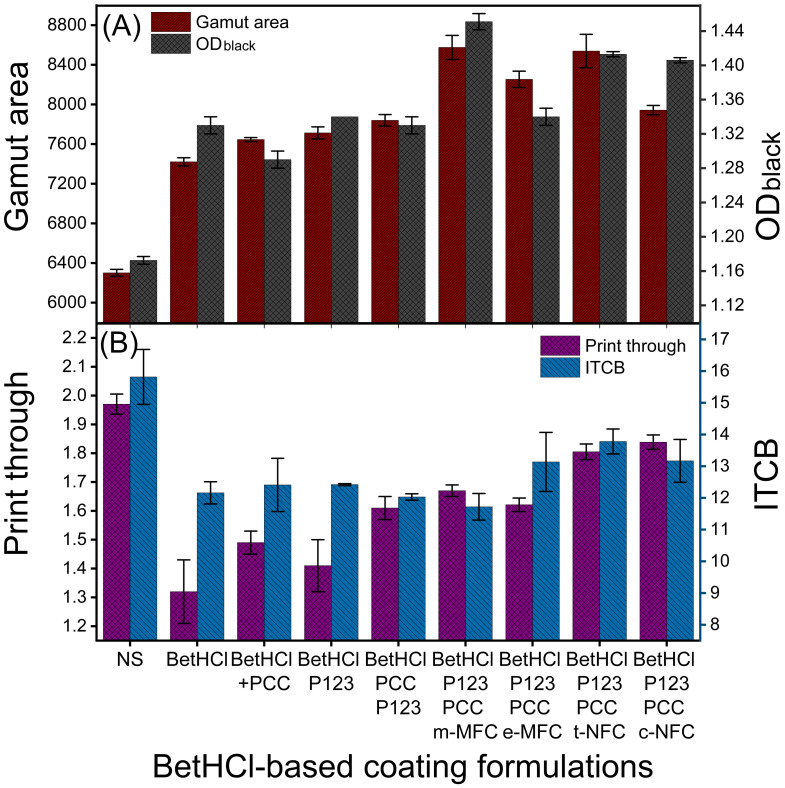
Effect of betaine hydrochloride on (**A**) gamut area, optical density for black, (**B**) print through and inter color bleed in presence of PCC, P123 and micro-/nanofibrillated celluloses.

As shown in Figure 6A, the addition of 16% of BetHCl improved the GA by 17.8%, whereas it increased by 21.3% in combination with PCC (16%), and by 22.4% with P123 (besides BetHCl). The combination of all these components improved the GA by 24.4%. The further addition of m-MFC (16%), along with the aforementioned agents, improved the GA by 36.1%, the highest increase among all the formulations in this study. Likewise, GA improved by 31.0%, 35.5% and 26.1% with the use of e-MFC, t-NFC and c-NFC (16%), respectively.

Similar to the GA, as shown in Table 8, gamut volume (GV) also improved by 25% with the addition of 16% BetHCl in compared to the reference formulation. In the further addition of 16% PCC along with the BetHCl in the formulation, there was an increase in the GV from 132 × 10^3^ to 168 × 10^3^, which corresponds to an increase of 27%, compared to the reference formulation. With the addition of 5% P123 along with the BetHCl, GV increased by 31%. When 16% PCC and 5% P123 were added along with the BetHCl in the formulation, it showed an increase of 34%.

The addition of m-MFC (in presence of PCC, P123 and BetHCl) resulted in an increase in GV from 132 × 10^3^ to 202 × 10^3^, which corresponds to an increase of 53%, compared to the reference formulation. The formulations, which correspond to incorporations of e-MFC, t-NFC and c-NFC showed increases in GV values from 132 (reference formulation) to 188, 198 and 183, corresponding to increments of 42%, 50% and 39%, respectively. Thus, as observed for the GA, there was also an increase in GV values with the addition of BetHCl, and comparable or even higher increments in gamut volume by including components such as PCC, P123 and M/NFCs.

Figure 6A also represents the OD (black) of BetHCl-coated papers. A 16% addition of BetHCl improved the OD by 13.7%, whereas it increased only by 10.3% with the BetHCl/PCC (16%) system, and by 14.5% when P123 (5%) was used in combination with BetHCl. Together, the three of them improved the OD (black) by 13.7%. Therefore, it may be concluded that PCC exerts either a negative influence or no significant influence on OD. By the further addition of m-MFC (16%), OD (black) improved by 24%, which was once again the highest improvement out of all the formulations that are reported herein. OD (black) increased by 14.5%, 20.8% and 20.2% with the use of e-MFC, t-NFC and c-NFC, respectively.

As shown in the Figure 6B, PT decreased by 33% (desired) with the addition of 16% of BetHCl. Moreover, with the addition of PCC and P123, PT decreased by 24 and 28%, respectively in combination of BetHCl. It was decreased by 18% with both PCC and P123, in a formulation with BetHCl. Similarly, it was decreased by 15, 18, 8 and 7% with m-MFC, e-MFC, t-NFC and c-NFC, respectively, in combination with BetHCl, PCC and P123. Thus, the highest improvement was observed with the use of BetHCl alone in the formulation with starch, OBA and AKD.

It was also shown in the Figure 6B that the ITCB improved by 23% with the addition of 16% of BetHCl in the coating formulation. Additionally, ITCB improved with 22 and 21% with the addition of PCC and P123, respectively, in the presence of BetHCl in the formulations. The presence of both PCC and P123 in a formulation improved the ITCB by 24% in combination with BetHCl. A highest improvement of 26% was observed with m-MFC in combination with BetHCl, PCC and P123, however, it was 17, 13 and 17% with the e-MFC, t-NFC and c-NFC, respectively.

Similar to SB coatings, Table 8 shows that the whiteness improved with the use of BetHCl; thus, these was no quenching of OBA in presence of these cationic substances in the coating formulations. Additionally, it increased by 3.7% with the used of BetHCl, PCC and P123 in the coating formulation. The presence of MFCs retained the whiteness, however, it substantially reduced with the use of c-NFC in the coating formulation. The Table 8 also represents the variations in Bendtsen roughness and Gurley air permeance, as also expected, without any significant impact.

### 3.3. Water Penetration Profile

**Figure 7:**
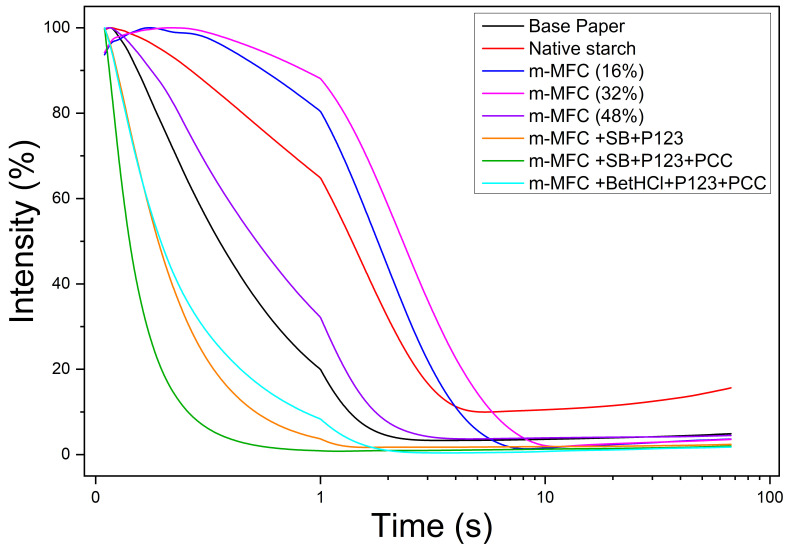
Penetration dynamic profile for mechanically produced MFC-coated paper.

Among all M/NFCs, m-MFC allowed for the best printing properties, particularly in terms of GA and OD. Besides displaying the best performance for that task, it should be remarked that the disc refiner approach is, according to previous works on the techno-economic assessment of M/NFCs [32], more industrially feasible. Hence, the choice of M/NFCs to monitor the water penetration profile was limited to m-MFC-based coatings, as shown in Figure 7. A useful parameter to establish insightful comparisons is t95, the time at which ultrasonic intensity decreases to 95% of the maximum value. This timespan, t95, was higher for native starch-coated paper (0.29 s) than for BP (0.14 s), as expected (Li et al., 2014 [33]). The sizing degree was further improved using 16% (t95 = 0.59 s) and 32% (t95 = 0.72 s) of m-MFCs. Above 32%, the sizing degree decreased as coating formulations showed less homogeneity (for 48% m-MFC, t95 = 0.37 s), as discussed previously, when assessing the rheological behavior. On the other hand, the sizing degree was reduced with the use of SB, P123, PCC and BetHCl [34]. This reduction is mostly due to the presence of P123, as evidenced by the lower SWCA for all P123-containing coating formulations and by the fast sorption rate as measured by DCA (see Figures S1 and S2 in the Supplementary Material).

## 4. Conclusions

The potential of using M/NFCs, starch betainate, betaine hydrochloride, Pluronics and PCC for low weight gain coatings and their effects on printing quality were investigated. The increasing concentration of M/NFCs improved the printing properties, but rheology and water penetration profile results allow one to suggest that M/NFCs should account for 16% of total solids, as higher concentrations affected the homogeneity of the formulations. Gamut area was improved by 19.2% (m-MFC), 17.7% (e-MFC), 20.3% (t-NFC) and 17.2% (c-NFC) when formulations were prepared using combinations of these M/NFCs with SB/Pluronics-P123 and PCC (16% each). However, c-NFC reduced the whiteness of the paper because of OBA quenching.

BetHCl-based coating improved the printing quality, as the gamut area increased by 36.1% (m-MFCs), 31.0% (e-MFC), 35.5% (t-NFC) and 26.1% (c-NFC) when formulations were prepared using a combination of these M/NFCs with Pluronics-P123 (5%), BetHCl and PCC (16% each). Likewise, the optical density for black color also improved by 24.0% (m-MFCs), 14.5% (e-MFC), 20.8% (t-NFC) and 20.2% (c-NFC) using said BetHCl-based coatings. Remarkably, despite containing the cationic groups that likely caused quenching when c-NFC was used, a similar loss in whiteness was not found for the Zwitterionic molecule BetHCl.

Among all the M/NFCs, m-MFC demonstrated the most favorable properties for coatings and, along with SB/P123/PCC and BetHCl, improved key printing parameters. Since the production of the other M/NFCs shared the homogenization step, which requires a large energy input, m-MFC seems to be the best choice out of the kinds of micro-/nanocelluloses that are reported here, both in terms of printing quality and industrial feasibility. In contrast, chemical and enzymatic pretreatments did not demonstrate convincing advantages, at least in what pertains to the improvement of the inkjet printing quality of office papers.

## 5. Patents

Provisional Portuguese patent application number-20221000002940 (28 July 2022-17:09:49).

## Figures and Tables

**Table 1:** Properties of micro-/nanofibrillated celluloses.

M/NFCs	Nano-Fibrillation Yield (%)	Zeta Potential (mV)	Intrinsic Viscosity (mL/g)	Degree of Polymerization
m-MFC	17	−24 ± 1	699	1869
e-MFC	2.5	−12 ± 1	664	1747
t-NFC	100	−45 ± 2	130	309
c-NFC	98	+27 ± 2	20	244

**Table 2:** Composition of coating components for micro-/nanofibrillated celluloses coatings, expressed as %*w*/*w*, on the basis of dry coating weight (each formulation contained 6% OBA and 0.4% AKD).

Coating Components	Concentration (% *w*/*w*)			
	Reference: 93.6% NS, 6% OBA, 0.4% AKD			
m-MFC	16/32/48	-	-	-
e-MFC	-	16/32/48	-	-
t-NFC	-	-	16/32/48	-
c-NFC	-	-	-	16/32/48
NS		77.6/61.6/45.6		

**Table 3:** Composition of coating components for combined coatings, expressed as %*w*/*w*, on the basis of dry coating weight (each formulation contained 6% OBA and 0.4% AKD).

Coating Components	Concentration (*w*/*w*%)							
	Reference: 93.6% NS, 6% OBA, 0.4% AKD							
	**MFCs**				**NFCs**			
m-MFC/e-MFC	16	16	16	16	-	-	-	-
t-NFC/c-NFC	-	-	-	-	16	16	16	16
SB	-	8	8	16	-	8	8	16
P123	-	8	8	16	-	8	8	16
PCC	-	-	8	16	-	-	8	16
NS	77.6	61.6	53.6	29.6	77.6	61.6	53.6	29.6

**Table 4:** Composition of coating formulations with betaine hydrochloride, encompassing varying quantities of P123, PCC and micro-/nanofibrillated celluloses (each formulation contained 6% OBA and 0.4% AKD).

Coating Components	Concentration (*w*/*w*%)							
	Reference: 93.6% NS, 6% OBA, 0.4% AKD							
2cBetHCl	16	16	16	16	16	16	16	16
m-MFC	-	-	-	-	16	-	-	-
e-MFC	-	-	-	-	-	16	-	-
t-NFC	-	-	-	-	-	-	16	-
c-NFC	-	-	-	-	-	-	-	16
P123	-	-	5	5	5	5	5	5
PCC	-	16	-	16	16	16	16	16
NS	77.6	72.6	61.6	56.6	40.6	40.6	40.6	40.6

**Table 5:** Apparent viscosity of micro-/nanofibrillated celluloses-starch based formulations at 10 and 1000 s^−1^ shear rates.

M/NFCs Conc.(%)	Shear Viscosity (Pa.s * 10^3^)			
	m-MFC	e-MFC	t-NFC	c-NFC
16	3.21/0.17	0.88/0.14	9.81/0.26	1.34/0.10
24	6.51/0.19	4.69/0.22	14.16/0.33	6.41/0.14
32	14.26/0.32	5.66/0.22	15.73/0.45	2.29/0.10
48	27.27/0.44	10.61/0.16	23.01/0.62	10.73/0.18

**Table 6:** Properties of coated papers using different concentrations of micro-/nanofibrillated celluloses in starch-based coating formulations. The amplitude of the tolerance intervals is standard deviation.

Coating Components	Conc. (%)	Roughness (mL/min)	Air Permeance (μm Pa^−1^ s^−1^)	CA (º)	Whiteness	Gamut Volume/10${}^{3}$	Weight Gain (g/m${}^{2}$)
Reference (NS)		329 ± 13	7.2 ± 0.2	72 ± 1	163 ± 1	132 ± 1	1.8 ± 0.3
m-MFC	16	383 ± 26	7.3 ± 0.4	70 ± 2	164 ± 1	149 ± 2	1.9 ± 0.2
	32	389 ± 17	5.7 ± 0.3	64 ± 1	166 ± 1	156 ± 2	2.3 ± 0.2
	48	397 ± 15	8.0 ± 0.5	67 ± 1	167 ± 1	158 ± 1	2.3 ± 0.1
e-MFC	16	355 ± 11	9.2 ± 0.2	83 ± 2	164 ± 1	138 ± 1	2.2 ± 0.2
	32	330 ± 11	9.8 ± 0.1	71 ± 3	164 ± 3	141 ± 1	1.9 ± 0.1
	48	321 ± 34	9.7 ± 0.6	78 ± 3	164 ± 1	142 ± 1	2.0 ± 0.1
t-NFC	16	340 ± 13	7.8 ± 0.9	75 ± 1	166 ± 2	145 ± 6	2.9 ± 0.2
	32	405 ± 18	5.9 ± 0.5	72 ± 1	166 ± 1	152 ± 1	2.5 ± 0.4
	48	384 ± 20	7.1 ± 0.2	60 ± 1	164 ± 1	156 ± 1	2.2 ± 0.2
c-NFC	16	347 ± 10	9.4 ± 0.4	88 ± 2	138 ± 1	148 ± 1	1.7 ± 0.1
	32	356 ± 11	7.1 ± 0.2	73 ± 2	120 ± 2	148 ± 1	2.5 ± 0.1
	48	358 ± 12	7.7 ± 1.2	67 ± 1	119 ± 1	150 ± 1	2.5 ± 0.4

**Table 7:** Properties of coated papers using micro-/nanofibrillated celluloses in presence of Starch betainate, P123 and PCC in starch-based coating formulations.

Coating Components	Conc. (%)	Roughness (mL/min)	Air Permeance (μm Pa^−1^ s^−1^)	CA (º)	Whiteness	Gamut Volume/10${}^{3}$	Weight Gain (g/m${}^{2}$)
Reference (NS)		329 ± 13	7.2 ± 0.2	72 ± 1	163 ± 1	132 ± 1	1.8 ± 0.3
m-MFC + SB + P123 + PCC	16, 8, 8, 0	387 ± 17	7.8 ± 0.3	45 ± 1	165 ± 1	158 ± 1	2.3 ± 0.2
	16, 8, 8, 8	380 ± 26	8.4 ± 0.3	45 ± 1	164 ± 1	162 ± 1	2.3 ± 0.1
	16, 16, 16, 16	432 ± 10	8.7 ± 0.0	40 ± 2	165 ± 1	172 ± 1	2.8 ± 0.1
e-MFC + SB + P123 + PCC	16, 8, 8, 0	349 ± 6	9.3 ± 0.2	50 ± 3	164 ± 1	154 ± 1	2.3 ± 0.2
	16, 8, 8, 8	321 ± 13	8.4 ± 0.4	49 ± 1	166 ± 1	154 ± 1	2.8 ± 0.1
	16, 16, 16, 16	333 ± 18	9.1 ± 0.0	44 ± 3	165 ± 1	165 ± 1	2.7 ± 0.2
t-NFC + SB + P123 + PCC	16, 8, 8, 0	381 ± 21	8.9 ± 0.3	44 ± 1	163 ± 1	156 ± 1	2.4 ± 0.2
	16, 8, 8, 8	404 ± 12	7.3 ± 0.0	46 ± 1	165 ± 1	159 ± 1	2.5 ± 0.1
	16, 16, 16, 16	352 ± 25	8.2 ± 0.2	40 ± 1	163 ± 1	168 ± 2	2.6 ± 0.1
c-NFC + SB + P123 + PCC	16, 8, 8, 0	399 ± 16	6.5 ± 0.5	34 ± 2	125 ± 4	158 ± 2	2.8 ± 0.2
	16, 8, 8, 8	382 ± 11	7.6 ± 0.3	42 ± 1	138 ± 1	161 ± 3	2.5 ± 0.1
	16, 16, 16, 16	375 ± 7	8.4 ± 0.6	33 ± 1	131 ± 2	163 ± 1	2.7 ± 0.4

**Table 8:** Properties of coated papers using betaine hydrochloride in presence of PCC, P123 and micro-/nanofibrillated celluloses in starch-based coating formulations.

Coating Component	Conc. (%)	Roughness (mL/min)	Air Permeance (μm Pa^−1^ s^−1^)	CA (º)	Whiteness	Gamut Volume/10${}^{3}$	Weight Gain (g/m${}^{2}$)
BetHCl	16	352 ± 15	6.1 ± 0.6	88 ± 1	165 ± 1	165 ± 1	2.6 ± 0.1
BetHCl + PCC	16, 16	313 ± 18	6.3 ± 0.6	78 ± 1	164 ± 1	168 ± 1	2.5 ± 0.3
BetHCl + P123	16, 5	345 ± 49	4.3 ± 0.0	43 ± 1	168 ± 1	173 ± 1	2.7 ± 0.3
BetHCl + PCC + P123	16, 16, 5	343 ± 26	4.8 ± 1.0	41 ± 2	169 ± 1	177 ± 2	2.8 ± 0.2
BetHCl + PCC + P123 + m-MFC	16, 16, 5, 16	407 ± 16	6.3 ± 0.2	42 ± 2	168 ± 1	202 ± 3	2.5 ± 0.5
BetHCl + PCC + P123 + e-MFC	16, 16, 5, 16	346 ± 09	7.9 ± 0.2	32 ± 1	167 ± 1	188 ± 2	2.8 ± 0.1
BetHCl + PCC + P123 + t-NFC	16, 16, 5, 16	463 ± 60	6.8 ± 0.5	45 ± 1	164 ± 2	198 ± 4	2.5 ± 0.4
BetHCl + PCC + P123 + c-NFC	16, 16, 5, 16	374 ± 15	7.2 ± 0.1	48 ± 1	128 ± 2	183 ± 1	2.7 ± 0.1

## Data Availability

The data presented in this study are available upon reasonable request from the corresponding author.

## Supplementary Materials

The following supporting information can be downloaded at: https://www.mdpi.com/article/10.3390/nano12162853/s1, Figures S1: Dynamic contact angle for papers coated using different concentration (w/w %) of e-MFC (A), m-MFC (B), c-NFC (C) and t-NFC (D) for starch-based coatings; Figure S2: Dynamic contact angle for papers coated using different concentration (w/w %) of SB, Pluronics P123 and PCC for e-MFC (A), m-MFC (B), c-NFC (C) and t-NFC (D) starch-based coatings.

## Abbreviations

The following abbreviations are used in this manuscript:

M/NFCs	Micro-/nanofibrillated celluloses
m-MFC	Mechanically produced microfibrillated cellulose
e-MFC	Enzymatically produced microfibrillated cellulose
TEMPO	Tetramethylpiperidine N-oxyl radical
t-NFC	TEMPO-mediated oxidation produced nanofibrillated cellulose
c-NFC	Cationic nanofibrillated cellulose
PVA	Poly-vinyl alcohol
AKD	Alkyl ketene dimer
SB	Starch betainate
OBA	Optical brightening agent
BetHCl	Betaine hydrochloride
MeBetCl	Methyl betainate chloride
DMF	*N*,*N*-dimethylformamide
BP	Base paper
PCC	Precipitated calcium carbonate
GA	Gamut area
GV	Gamut volume
OD	Optical density
PT	Print-through
ITCB	Inter-color bleed
BEKP	Bleached eucalyptus kraft pulp
BPKP	Bleached pine kraft pulp
SWCA	Static contact angle with water
DWCA	Dynamic contact angle with water
