# Supplementary material for: Micro-/Nanofibrillated Cellulose-Based Coating Formulations: A Solution for Improving Paper Printing Quality
Source: Nanomaterials (Basel). 2022 Aug 18;12(16):2853. doi: 10.3390/nano12162853 (PMC9414902; doi:10.3390/nano12162853)
Supplement: Supplementary file 1 [file nanomaterials-12-02853-s001.zip › nanomaterials-1866128-supplementary.pdf]

Supplementary materials

Micro-/Nanofibrillated Cellulose-Based Coating Formulations: A Solution for Improving Paper Printing Quality

Mohit Sharma [1,*], Roberto Aguado [2], Dina Murtinho [3], Artur J. M. Valente[3] and Paulo J. T. Ferreira[1]

[1] University of Coimbra, CIEPQPF, Department of Chemical Engineering, Rua Sílvio Lima, Polo II–Pinhal de Marrocos, 3030-790 Coimbra, Portugal

[2] LEPAMAP-PRODIS Research Group, University of Girona, M Aurélia Capmany 61, 17003 Girona, Spain

[3] University of Coimbra, CQC, Department of Chemistry, Rua Larga, 3004-535 Coimbra, Portugal

* Correspondence: mohit@eq.uc.pt

This supplementary document contains dynamic water contact analysis (DWCA) results. Although regarded as supplementary, this information is necessary to support some claims given along the article.

S1 Dynamic water contact analysis (DCA)

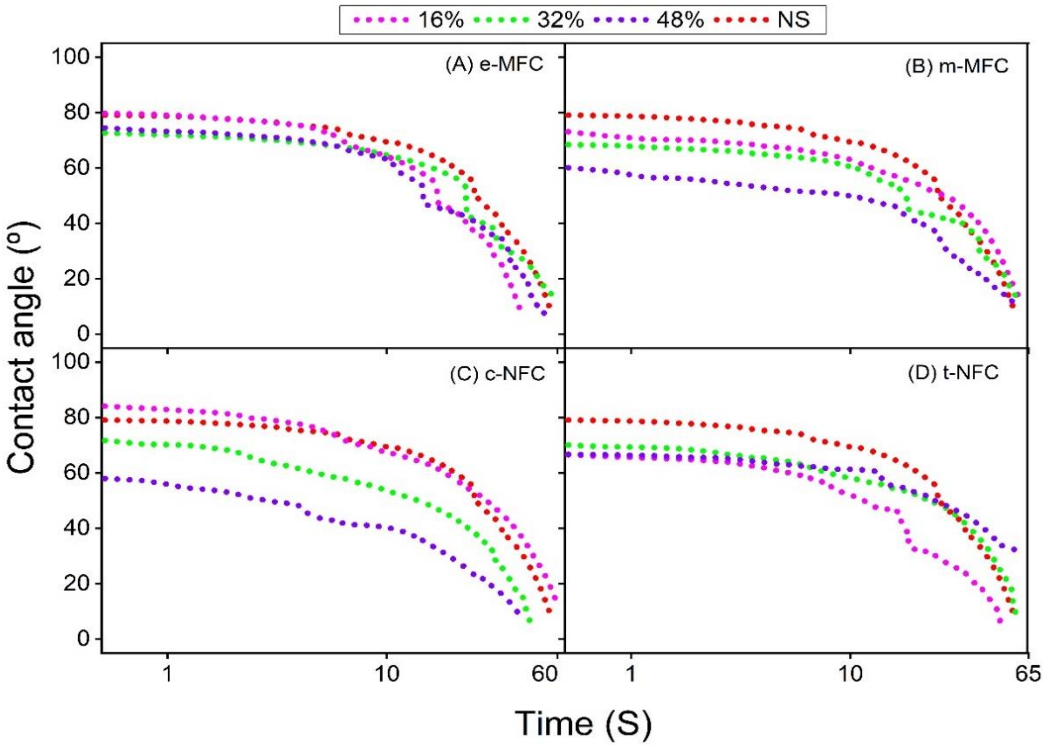

Figure S1. Dynamic contact angle for papers coated using different concentration (w/w %) of e-MFC (A), m-MFC (B), c-NFC (C) and t-NFC (D) for starch-based coatings

Figure S1 shows the DWCA plot, i.e., the time profile of the water contact angle, for M/NFCs coated samples (Refer Table 2 in main manuscript for detail of used concentrations). Likewise, DWCA of combined coatings

using M/NFCs/starch, Starch betainate (SB), Pluronics and PCC (Refer Table 3 in main manuscript for detail of used concentrations) is displayed in Figure S2.

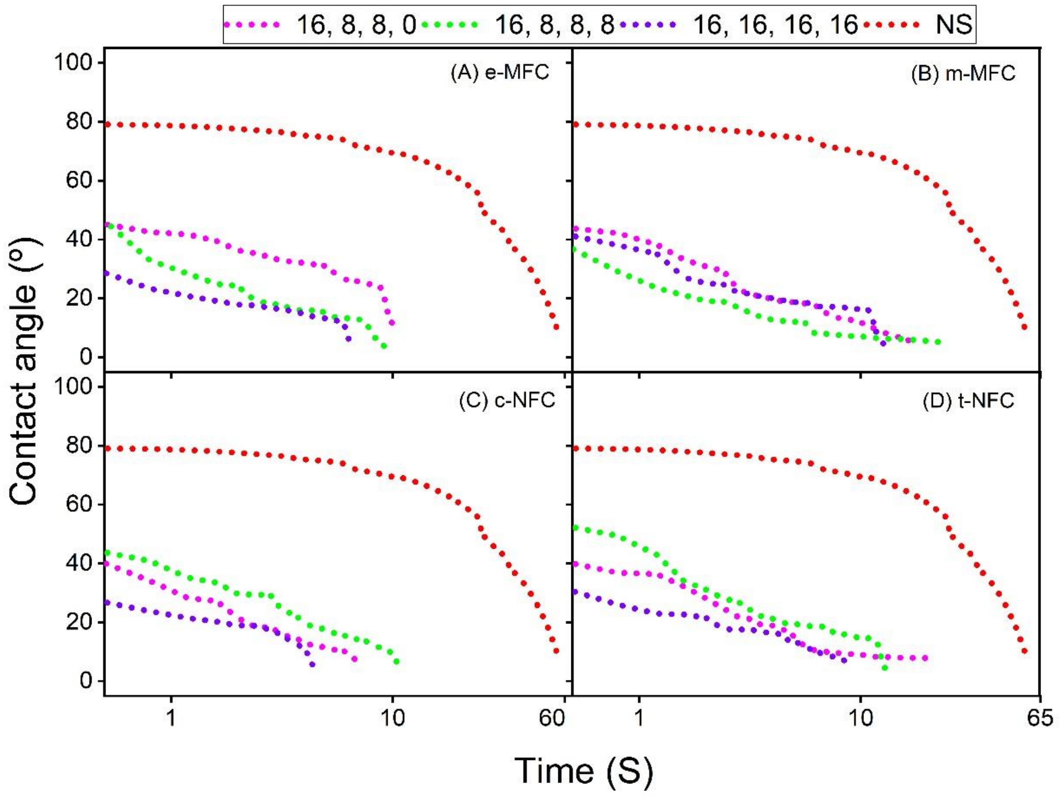

Figure S2. Dynamic contact angle for papers coated using different concentration (w/w %) of SB, Pluronics P123 and PCC for e-MFC (A), m-MFC (B), c-NFC (C) and t-NFC (D) starch-based coatings
